# Costs analysis of the treatment of imported malaria

**DOI:** 10.1186/1475-2875-11-1

**Published:** 2012-01-02

**Authors:** Viera Svihrova, Maria Szilagyiova, Elena Novakova, Jan Svihra, Henrieta Hudeckova

**Affiliations:** 1 Department of Public Health, Jessenius Faculty of Medicine Comenius University, Sklabinska 26, 037 53 Martin, Slovak Republic; 2 Clinic of Infectology and Travel Medicine, Jessenius Faculty of Medicine Comenius University, Kollarova 2, 036 59 Martin, Slovak Republic; 3 Department of Microbiology and Immunology, Jessenius Faculty of Medicine Comenius University, Sklabinska 26, 037 53 Martin, Slovak Republic; 4 Clinic of Urology, Jessenius Faculty of Medicine Comenius University, Kollarova 2, 036 59 Martin, Slovak Republic

**Keywords:** Imported malaria, Costs, Prevention

## Abstract

**Background:**

To document the status of imported malaria infections and estimate the costs of treating of patients hospitalized with the diagnosis of imported malaria in the Slovak Republic during 2003 to 2008.

**Case study:**

Calculating and comparing the direct and indirect costs of treatment of patients diagnosed with imported malaria (ICD-10: B50 - B54) who used and not used chemoprophylaxis. The target sample included 19 patients diagnosed with imported malaria from 2003 to 2008, with 11 whose treatment did not include chemoprophylaxis and eight whose treatment did.

**Results:**

The mean direct cost of malaria treatment for patients without chemoprophylaxis was 1,776.0 EUR, and the mean indirect cost 524.2 EUR. In patients with chemoprophylaxis the mean direct cost was 405.6 EUR, and the mean indirect cost 257.4 EUR.

**Conclusions:**

The analysis confirmed statistically-significant differences between the direct and indirect costs of treatment with and without chemoprophylaxis for patients with imported malaria.

## Background

Malaria is a common and life-threatening disease in areas where it is endemic. In the Slovak Republic, malaria is currently categorized as an imported infectious disease. In the past years, there was a balanced trend in the Slovak Republic in the numbers of reported cases of imported malaria, no deaths have been confirmed. The disease has traditionally been expensive to treat. Malaria chemoprophylaxis, a very effective protection against the infection, is important not only for health reasons but also because it reduces the costs of treating the disease and, in the case of working persons, it minimizes possible social-economic impacts on the patient and his/her family and society.

The goal of this paper was to calculate and compare the direct and indirect costs of treatment with and without chemoprophylaxis in patients diagnosed with imported malaria (ICD-10: B50 - B54). This is the first study in Slovakia about hospitalization costs of imported malaria.

## Methods

Data on imported malaria patients in the Slovak Republic from 2003 to 2008 were obtained from the Epidemiological Information System of the Office of the Public Health of the Slovak Republic (EPIS). Analysis has evaluated costs during hospitalization. Patients without hospitalization were observed in home surroundings, this presents zero costs for hospitalization. From the data on hospitalized patients, it was calculated and estimated the direct cost to health insurance companies for the treatment of all patients. The costs of hospitalization were obtained from the health insurance companies and from the Health Care Surveillance Authority. The 2008 EUR exchange rate was used for cost calculations. The direct costs of hospitalization and of laboratory and imaging examinations were included.

Indirect costs included those to employers in the form of compensation for lost income to the health funds of the Social Insurance Company and health benefits as well as the production losses due to the reduction of the gross domestic product (GDP) during the patients' working disability. During adult patients' first ten days of working disability, their income is compensated by the employer at a rate of 25% of the daily-calculated basis for the first three days and then at a rate of 55% [1]. The lowest possible calculated daily base is defined as one-thirtieth of the minimum wage for workers with a monthly wage on the day on which the valid claim for compensation was made [2]. For calculation of indirect costs, the gross wages of employees were used. By law, in the Slovak Republic, there are guaranteed gross minimal wages. In calculating the costs, this was used as a base the minimum wage in 2008, which was 268.871 EUR monthly or 1.547 EUR hourly [3].

All group data were expressed as the mean and the standard deviation (SD). The costs incurred by patients without chemoprophylaxis were compared with those incurred by patients with chemoprophylaxis using the Mann-Whitney *U *test. A p value less than 0.05 was accepted as the level of statistical significance. Data were processed using the SPSS software Windows edition, version 11.0.

## Results

During the study period 19 cases of imported malaria were identified by the EPIS in Slovak Republic. All of the above patients had visited areas where malaria was endemic; only eight (42%) of them had used malaria chemoprophylaxis before and during their sojourn in the endemic areas. Hospitalization was not required in two cases where chemoprophylaxis has been used, and the patients were treated as out-patients. Therefore, 17 patients were hospitalized with the diagnosis of imported malaria. Eleven patients had not used chemoprophylaxis; their mean age was 34 years, with a range of 20 - 55 years. The length of their hospitalization was 5 - 26 days, with a mean length of 13 days. Eight patients had used chemoprophylaxis; their mean age was 27 years, with a range 22 - 35 years. The length of their hospitalization was 0 - 11 days, with a mean length of 6.5 days (Table 1).

**Table 1:** Imported malaria in the Slovak Republic, 2003 - 2008

Case	Age	Gender	Place of acquisition	Plasmodium species	Length of inpatient stay	Using of antimalaria chemoprophylaxis
**2003**						
1	25	male	Chad	*P. falciparum*	15 days	no
2	42	male	Myanmar	*P. vivax*,	20 days	no
				*P. falciparum*		
3	27	male	Ivory Coast	*P. falciparum*	0 days	yes
4	24	female	South East Asia	*P. falciparum*	0 days	yes
**2004**						
5	26	male	Eritrea	*P.vivax*	11 days	yes
6	24	male	Eritrea	*P.vivax*	10 days	yes
7	22	male	Eritrea	*P.vivax*	10 days	yes
8	35	male	Eritrea	*P.vivax*	6 days	yes
**2005**						
9	32	male	Cameroon	*P. falciparum*	5 days	yes
**2006**						
10	55	female	Uganda	*P. vivax*	10 days	no
11	27	male	Ivory Coast	*P. vivax*	10 days	yes
12	34	male	Equatorial Guinea	*P. falciparum*	15 days	no
13	35	male	Benin	*P. falciparum*	26 days	no
14	20	male	Benin	*P. falciparum*	14 days	no
15	28	male	Ecuador	*P. vivax*,	9 days	no
				*P. falciparum*		
16	41	male	Angola	*P. falciparum*	11 days	no
**2007**						
17	32	male	Equatorial Guinea	*P. falciparum*	5 days	no
**2008**						
18	20	male	Ghana	*P. falciparum*	10 days	no
19	37	male	Namibia, S.Africa	*P. falciparum*	9 days	no

Most infections were acquired in Africa (17 cases - 89%). The infectious agents were *Plasmodium vivax *in six cases (35%) and *Plasmodium falciparum *in eleven cases (53%). Two patients (12%) had dual infections. Uncomplicated versus complicated diseases were found in 16 versus three cases (2× *Plasmodium falciparum*, 1× dual infection).

After calculating the costs of hospitalization to the health insurance company, the mean direct cost of treatment for patients without chemoprophylaxis was 1,776.0 EUR (527.1 - 7,029.8 EUR). In patients with chemoprophylaxis, the mean direct costs per patient was 405.6 EUR (0.0 - 543.5 EUR) (Table 2).

**Table 2:** Comparison of direct and indirect costs in EUR of imported malaria with and without chemoprophylaxis

	n without chemoprophylaxis		n with chemoprophylaxis		
**n = 11 without chemoprophylaxis**	**mean**	**SD**	**mean**	**SD**	**p values**

**direct costs**					
price of hospitalization	1419.7	2121.9	349.5	215.7	< 0.05
price of laboratory and imaging examinations	356.3	430.8	56.1	35.1	< 0.05
**total price**	**1776.0**	**2518.5**	**405.6**	**250.4**	< 0.05
**indirect costs**					
compensation of income by employer and social insurance	78.0	40.0	35.9	26.4	< 0.05
loss of GDP	446.2	200.2	221.6	154.6	< 0.05
**total indirect costs**	**524.2**	**240.2**	**257.4**	**180.9**	< 0.05
**total direct and indirect costs**	**2300.2**	**2669.4**	**663.0**	**419.0**	< 0.05

loss of income during inoperable	84.0	32.7	44.6	29.9	< 0.05

The indirect costs included compensation of loss of income by the employer for the first ten days of the working disability and by the Social Insurance Company thereafter. In patients without chemoprophylaxis, the income compensation by employer and social insurance represented the sum of 78.0 EUR for 13 days of working disability, which mean a 52% decrease in income.

Other indirect costs are not negligible: lost salaries decreased production and lowered GDP. For patients without chemoprophylaxis, the mean loss of income was 84.0 EUR (39,0 - 156,0 EUR), and the mean loss of productivity at the current GDP was 446.2 EUR (170.4 - 886.2 EUR). In patients with chemoprophylaxis, the mean loss of income was 44.6 EUR (0.0 - 72.4 EUR), and the mean loss of GDP was 221.6 EUR (0.0 - 374.9 EUR). The total indirect costs for patients without and with chemoprophylaxis were 524.2 EUR (193.3 - 1,052.0 EUR) and 257.4 EUR (0.0 - 438.6 EUR), respectively.

## Discussion

**Figure 1:**
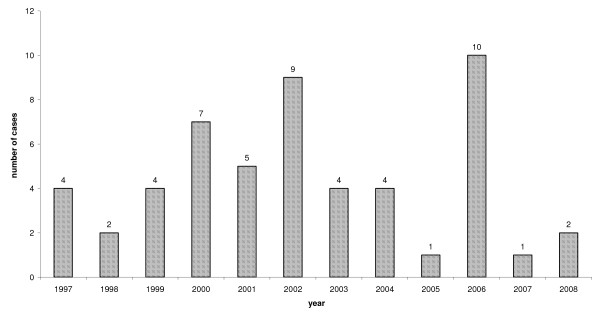
**Reported cases of imported malaria in the Slovak Republic (B50 - B54) 1997-2008**.

Malaria is currently endemic in over 100 countries, which are visited by more than 125 million international travellers every year. Each year, many international travellers fall ill with malaria while visiting these countries and well over 10,000 is reported to fall ill after returning home. Due to underreporting, the real figure may be as high as 30,000 [4]. During the years 2008-2009, 25 cases of imported malaria were registered in Romania, with no fatalities [5]. The most significant endemic areas for malaria are in Sub-Saharan Africa, the South-West Pacific, South-East Asia and the rainforests of South America [6]. According to the EPIS, 53 cases of imported malaria were reported in the Slovak Republic during 1997 to 2008 (Figure 1), with a decreasing trend similar to the decreases in the Netherlands and the UK [7,8].

According to the Centers for Disease Control and Prevention, the annual incidence of malaria in the world accounts for 190-311 million clinical episodes and 708,000 - 1,003,000 deaths [9]. According to many scholars, the disease causes as much as 0.6% - 1.3% loss of the GDP in countries with high malaria incidences [10,11]. In these countries, the costs of treating one case of malaria are 1.4 USD in Ethiopia, 6.3 USD in Sudan, and 8.0 USD in Burkina Faso [12-14]. In two provinces of Papua New Guinea total mean inpatient malaria episode costs were 25.2 USD in Madang and 14.1 USD in Maprik [15].

In the available literature, only three comprehensive calculations of the direct and indirect costs of imported malaria in high-income countries were found. The costs in patients from Switzerland and the Federal German Republic were lower for those who had undergone mefloquine prophylaxis than for those who had not [16]. In the UK, the costs of treating malaria without chemoprophylaxis greatly exceed the costs of chemoprophylaxis, showing the prophylaxis to be highly cost effective. This is clearly shown by the cost-benefit ratios. Except for malaria, the benefits (expressed as avoided costs) did not exceed the incurred costs [17]. Keystone showed that the costs of pre-travel consultations and of inexpensive vaccines and malaria prophylaxis would likely be easily offset by the savings owing to reduced health care costs incurred from the treatment of imported infectious diseases [18]. In these cases, prevention is better than treatment.

One study analysed the case of fifteen soldiers from the British army who required intensive hospital therapy because of malaria infection. Out of 24,600 British troops stationed in Germany, approximately 800 were occupationally exposed to malaria during 2001 and 800 during 2002. Three imported malaria cases were reported in British soldiers during 2001 and 12 during 2002. Two soldiers, one with *P. vivax *and the other with *P. falciparum *infections. required intensive hospital therapy. The median length of patient stay in hospital was seven days for a *P. vivax *infection, and 8.5 days for a *P. falciparum *infection. The direct treatment costs of the hospitalizations totalled 27,760 EUR [19]. All soldiers in this study were prescribed mefloquine for malaria chemoprophylaxis.

It is generally known that no anti-malarial prophylactic regimen gives complete protection, but good chemoprophylaxis (adherence to the recommended drug regimen) does reduce the risk of fatal disease [4]. Malaria chemoprophylaxis is unequivocally cheaper than the treatment of malaria. In this series, statistically-significant differences between the direct and indirect costs among patients who had imported malaria, with and without chemoprophylaxis, were confirmed. All anti-malarial drugs have specific contraindications and possible side effects. Adverse reactions attributed to malaria chemoprophylaxis are common, but most are minor and do not affect the activities of the traveller. Depending on the malaria risk in the area visited, the recommended prevention method may only be mosquito bite prevention, or mosquito bite prevention in combination with chemoprophylaxis [4]. When mefloquine was used, it represented 4.7% of the total direct costs of treatment in Slovak Republic of patients with the lowest compensation from the health insurance company. The costs of two months of prevention by mefloquine chemoprophylaxis (24.8 EUR) are lower than the cost of lost income from a five-day working disability (39.0 EUR). In the case of patient with the highest direct costs the use of chemoprophylaxis represented only 0.4% of the total direct costs for the treatment [20].

This is the first analysis of the costs of hospitalization for imported malaria in Slovakia. The results confirmed that the prevention of malaria is worthwhile. Prevention decreases the occurrence of disease, reduces process of disease and reduces costs for hospitalization. In Slovakia, this disease is rare and the number of cases is limited. Trends show that number of cases is not increasing and death had not been confirmed in previous years.

## Conclusions

The continuous growth of professional and leisure travel to malaria-endemic regions may lead to an increase of imported cases, especially if prophylactic measures are not strictly followed. In the Slovak Republic, malaria chemoprophylaxis is not reimbursed by health insurance. The analysis by Pistone et al. shows that a policy change toward reimbursing malaria chemoprophylaxis for travellers from France to sub-Saharan Africa would be cost-effective for the European health insurance system [21]. A similar policy should be considered in the Slovak Republic.

## Competing interests

The authors declare that they have no competing interests.

## Authors' contributions

VS identified literature sources, conceived acquisition of data, participated in the sequence alignment and drafted the manuscript. MS conceived of the study, and participated in its design and coordination and helped to draft the manuscript. EN participated in the interpretation of data. JS participated in the design of the study and performed the statistical analysis and contributed to the writing of the manuscript. HH conceived of the study, and participated in its design and coordination and helped to draft the manuscript. All authors read and approved the final manuscript.
